# Linoleum—A Great Discovery

**Published:** 1874-11

**Authors:** 


### LINOLEUM—A GREAT DISCOVERY.

A custom which prevails all over the country is that of placing a bit of oil-cloth in front of the wash-bowl in sleeping apartments, to catch any drops which may chance to fall in the course of the morning's ablutions. We have always looked upon it as an abominable custom, and have inveighed against it ever since we can remember. We step on it with bare feet and are chilled to the soul. We appreciate the fact that it is waterproof, and that it saves the carpet underneath, but we wonder why some substitute cannot be found, which, while repelling moisture, will not extract the last atom of caloric from the feet exposed to it.

We believe we have found the substitute at last. It comes in the form of a carpet-stuff, made by grinding cork—one of the best non-conductors in the world—mixing it with linseed oil, and spreading the mass, by very heavy pressure, between powerful rollers upon canvass. The product is called by the makers, "Linoleum," which is simply the Latin term for linseed oil.

The cost of this carpet is about the same as the best painted carpeting, or oil-cloth. Its natural color is a soft brown, upon which a multitude of chaste designs are imprinted in the usual way. It makes a very graceful and elegant floor covering, is noiseless to the tread, impervious to moisture, and accumulates no dust. It is laid with cement, and exhibits no joints. It does not shrink and swell and bulge and crack, as oil-cloth is sure to do sooner or later. It is said to outwear by far, the best painted carpets manufactured.

But looked at in a hygienic point of view—the view we are bound to take of it—it is a great boon to the human race. It is a non-conductor, a non-absorbent of caloric, and its temperature is about the same in mid-winter as in mid-summer. You may stand on it all day with your thin-soled slippers, and your feet and lower extremities will not suffer from the contact. Cover your office floor with it, and you will not go forth with cold feet and a congested brain. Spread it upon your dining-room, and the mildest degree of warmth will be sufficient for your comfort, because there is no extractor of caloric constantly at work beneath, to chill your bodies. Bridget in the kitchen or laundry can not complain of colds and headaches all winter, because the blood circulates freely, and is not driven from the lower limbs to the vital organs.

We are delighted with the new carpeting, and bespeak for it a place in every house. If it could supersede woolen carpets, those disease-producing creations which catch dust only to give it off to the lungs at every step, it would work incomparable good. This new carpeting is certain to be largely used in all places where noise is to be avoided, such as hospitals, asylums, school-rooms, banks, and offices; and as the feet do not readily slip upon it, its employment in steamers and in palace cars, will work a great advantage. Up to a very recent period, England has supplied the market of America with this admirable floor covering, but a company formed, we believe, by English and American capital, have recently commenced its manufacture here. They have purchased a large tract of land on Staten Island, have erected suitable works, and are producing carpeting superior in all respects to the British product. If this wholesome and delightful article does not ultimately supercede the old oil-cloth, we shall be mistaken.

